# A brood parasite selects for its own egg traits

**DOI:** 10.1098/rsbl.2013.0573

**Published:** 2013-10-23

**Authors:** Claire N. Spottiswoode

**Affiliations:** 1 Department of Zoology, University of Cambridge, Downing Street, Cambridge CB2 3EJ, UK; 2 Percy FitzPatrick Institute, DST/NRF Centre of Excellence, University of Cape Town, Rondebosch 7701, South Africa

**Keywords:** coevolution, mimicry, intraspecific competition, superparasitism, virulence

## Abstract

Many brood parasitic birds lay eggs that mimic their hosts' eggs in appearance. This typically arises from selection from discriminating hosts that reject eggs which differ from their own. However, selection on parasitic eggs may also arise from parasites themselves, because it should pay a laying parasitic female to detect and destroy another parasitic egg previously laid in the same host nest by a different female. In this study, I experimentally test the source of selection on greater honeyguide (*Indicator indicator*) egg size and shape, which is correlated with that of its several host species, all of which breed in dark holes. Its commonest host species did not discriminate against experimental eggs that differed from their own in size and shape, but laying female honeyguides preferentially punctured experimental eggs more than host or control eggs. This should improve offspring survival given that multiple parasitism by this species is common, and that honeyguide chicks kill all other nest occupants. Hence, selection on egg size in greater honeyguides parasitizing bee-eaters appears to be imposed not by host defences but by interference competition among parasites themselves.

## 1. Introduction

Egg mimicry by cuckoos is a textbook example of coevolution, arising from selection by discriminating hosts that reject mismatched eggs from their nests [1]. However, a non-mutually exclusive alternative hypothesis is that cuckoos themselves impose selection on eggs [2]: it should pay a laying cuckoo to detect and destroy an egg laid by another parasitic female, lest it kill or outcompete her own chick were it to hatch first. Tests to date of this hypothesis in common cuckoos *Cuculus canorus* [2] and Horsfield's bronze-cuckoos *Chalcites basalis* [3] have revealed no clear evidence in support.

Egg discrimination by parasites should be most likely to evolve when multiple parasitism (or ‘superparasitism’: more than one parasitic female laying in the same host nest) is common. This is so in the greater honeyguide *Indicator indicator*, an African brood parasite whose chick obligately kills its nest-mates [4]: in my study population in Zambia, 35% of parasitized nests where the final clutch composition was known (*n* = 40) contained more than one parasitic egg, compared with 11% and 3%, respectively, in the cuckoo species above. This might result from the apparent lack of territoriality of greater honeyguide females [5].

Greater honeyguide eggs differ in size and shape in the nests of different species, correlated to the size and shape of host eggs but remaining on average larger than them [6]. This phenotypic differentiation corresponds partially to two ancient genetic matrilines specializing on hosts in each of two nest types [6]. Parasitic eggs are always white, unlike those of some of their hosts, but egg size and shape may be more informative cues of identity in the darkness of the tree cavities or deep burrows in which hosts breed [6]. However, the possible adaptive role of this specialization remains unknown.

This study experimentally tests the source of selection on greater honeyguide egg size in nests of its commonest host, the little bee-eater *Merops pusillus*. First, I establish that hosts do not reject or desert foreign eggs differing from their own in size and shape. I then test the hypothesis that honeyguide females themselves impose selection on egg dimensions by eliminating potential competitors. A possible mechanism is egg-puncturing behaviour: like certain other brood parasitic birds [1,7], greater honeyguides typically make 1–37 (mean 7.3) punctures in host eggs when laying their own [8]. Embryo mortality increases with puncture quantity, and lightly punctured eggs sometimes hatch viable offspring [4,8]. Honeyguides puncture more heavily when laying late relative to the host, suggesting they can strategically adjust puncturing behaviour in relation to its potential benefits [8]. I therefore test the prediction that laying female honeyguides preferentially puncture potentially parasitic eggs.

## 2. Material and methods

Data were collected within a ca 40 km^2^ area centred on Musumanene Farm (16°47′ S, 26°54′ E), Choma, Zambia, during September–November 2008–2011. Bee-eaters nest in deep subterranean burrows that we excavated then reconstructed at each visit [4]. About two-thirds of little bee-eater breeding attempts were visited by at least one greater honeyguide [4].

To test whether hosts reacted defensively to a foreign egg, I added an experimental egg to unparasitized host clutches, usually before or shortly after clutch completion (three to six eggs). This mimics the laying behaviour of real honeyguides, which do not remove host eggs and lay any time during host incubation [4]. For logistical and ethical reasons, it was impractical to use real honeyguide eggs. Instead, experimental eggs were unmarked white or off-white eggs of other species, larger than host eggs and differing in shape from host and honeyguide eggs to varying degrees (table 1). Medium-sized experimental eggs roughly corresponded in size to eggs laid by greater honeyguides specializing on larger hosts, whereas the largest eggs were larger than any greater honeyguide eggs at this site [6]. Eggs were considered accepted if they remained in the host nest for more than 3 days. No egg accepted at 3 days was subsequently rejected.

**Table 1.** Dimensions and thickness of host, parasite and experimental eggs. One host or experimental egg per clutch was analysed to avoid pseudoreplication.

	length (mm)	breadth (mm)	volume (cm^3^)	shape	thickness^a^ (mm)	*n* (thickness)	*n* (host reaction)	*n* (parasite reaction)
little bee-eater (host) *Merops pusillus* (*n* = 186 clutches)	18.14 ± 0.05 (16.32–21.00)	15.48 ± 0.03 (14.43–16.73)	2.22 ± 0.01 (1.84–2.91)	0.85 ± 0.00 (0.78–0.93)	0.072 ± 0.001 (0.062–0.090)	41		
greater honeyguide (parasite) *Indicator indicator* (*n* = 113)	22.83 ± 0.08 (20.62–24.78)	17.69 ± 0.05 (16.05–19.28)	3.65 ± 0.03 (2.75–4.53)	0.78 ± 0.00 (0.68–0.86)	0.126 ± 0.002 (0.107–0.145)	41		
experimental								
ring-necked dove *Streptopelia capicola*	26.90 ± 0.32 (24.67–29.37)	20.89 ± 0.22 (19.75–22.79)	6.01 ± 0.18 (5.20–7.51)	0.78 ± 0.01 (0.73–0.83)	0.117 ± 0.003 (0.103–0.135)	10	11	8
emerald-spotted wood-dove *Turtur chalcospilos*	23.49 ± 0.18 (22.23–25.38)	17.40 ± 0.12 (16.01–18.30)	3.63 ± 0.07 (2.92–4.25)	0.74 ± 0.01 (0.67–0.78)	0.093 ± 0.002 (0.081–0.102)	10	19	5
laughing dove *Streptopelia senegalensis*	24.75 ± 0.47 (23.46–25.63)	19.65 ± 0.57 (18.13–20.83)	4.90 ± 0.36 (3.93–5.57)	0.79 ± 0.01 (0.77–0.83)			4	0
Namaqua dove *Oena capensis*	20.79 ± 0.45 (20.34–21.23)	15.85 ± 0.10 (15.75–15.94)	2.66 ± 0.09 (2.57–2.75)	0.76 ± 0.01 (0.75–0.77)	0.079 ± 0.002 (0.073–0.088)	10	1	1
chestnut-bellied kingfisher *Halycon leucocephala*	24.29 ± 0.12 (22.99–25.08)	21.30 ± 0.09 (20.70–22.08)	5.62 ± 0.07 (5.02–6.10)	0.88 ± 0.00 (0.84–0.90)	0.079 ± 0.002 (0.071–0.089)	10	10	12
golden-tailed woodpecker *Campethera abigoni*	22.74 ± 0.11 (22.63–22.84)	18.13 ± 0.04 (18.09–18.16)	3.81 ± 0.03 (3.78–3.84)	0.80 ± 0.00 (0.80)	0.156 ± 0.004^b^ (0.152–0.161)	4	1	1

^a^Data sources for thickness: egg collection of J. F. R. Colebrook-Robjent, Zambia, except ^b^Natural History Museum, Tring, UK (for which *n* = 2 clutches only).

To test whether laying honeyguides preferentially punctured experimental eggs, I added an egg as soon as possible after clutch initiation to pre-empt real parasitism (sample sizes in table 1). In 17 of the 27 trials visited by a honeyguide, a control egg (a little bee-eater egg from another nest) was exchanged for a host egg, in case hosts use some other cue to detect foreign eggs. Some trials also contributed to the previous experiment if honeyguide parasitism occurred after more than 3 days. Nests were revisited every 3–5 days. If parasitized, the number of punctures and independent cracks in each egg was counted. If either a host (*n* = 13) or an experimental (*n* = 12) egg was completely destroyed or so badly cracked open that counting punctures and cracks was impossible, it was arbitrarily assigned the maximum recorded in a bee-eater egg (*n* = 37 punctures [8]). (Heavily damaged eggs usually remained in the nest but if no longer whole were usually removed by hosts.) This assumption is conservative because host eggs were smaller than experimental eggs. Bigger eggs may be punctured more simply because a honeyguide puncturing at random in a dark nest chamber may have a higher chance of hitting a larger egg. Therefore, I conservatively analysed punctures per unit surface area. Volume and surface area were calculated following [9,10].

Shell thickness could affect puncturing behaviour, if it influences puncture resistance or gives honeyguides a cue to egg identity. Species-specific data were obtained by directly measuring shell thickness for one egg per clutch in museum collections, following [8], and entered as a covariate (table 1).

Punctures per unit area (punctures + 1 divided by surface area) was log-transformed before analysis. Egg identity (host, control or experimental) and shell thickness of the experimental egg were modelled as fixed effects, and clutch membership (and thus honeyguide laying event) as a random effect.

## 3. Results

### (a) Multiple parasitism by honeyguides

Where two honeyguide eggs were laid in a host nest, they differed more in volume from one another than from randomly sampled honeyguide eggs (intraclass correlation coefficient = −0.22, *p* = 0.81, *n* = 32 eggs in 16 nests), supporting the assumption that different females laid them [11]. Considering only eggs with known fates, no first-laid honeyguide egg (*n* = 18) in a multiply parasitized nest ever survived close to hatching, compared with 25% of second-laid eggs (*n* = 16; Fisher's exact test, *p* = 0.039). The latter resembled the success rate of honeyguide eggs in nests parasitized once only (30%; *n* = 63, *p* = 0.77). The main cause of honeyguide hatching failure was desertion (see also [4]).

### (b) Do bee-eaters react to foreign eggs?

Experimental eggs were accepted in 45 of 46 nests. Experimental eggs were known to survive for 3–23 days (mean 8.5), depending on monitoring rate and whether a honeyguide subsequently visited. In the single nest where the experimental (ring-necked dove) egg was rejected, it lay intact below the nest entrance.

### (c) Do honeyguides react to foreign eggs?

**Figure 1.**
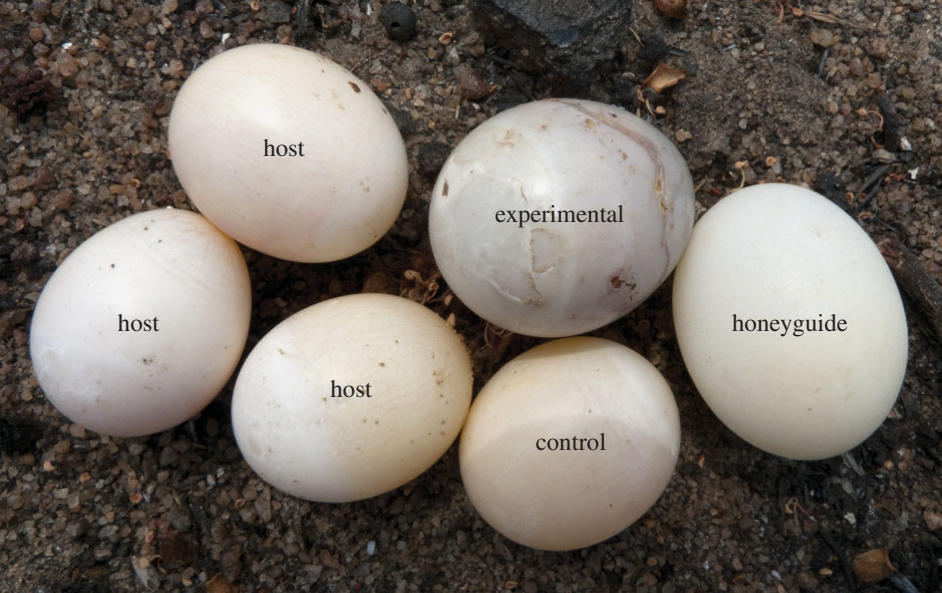
Example of an experimentally manipulated clutch after parasitism by a greater honeyguide (nest 2010_MP051, 27 October 2010). (Online version in colour.)

Honeyguides visited 27 manipulated nests. In 21 nests, at least one honeyguide egg was laid (figure 1; one egg in 16 cases and two eggs in five cases, when punctures were counted before the second honeyguide laid). In the remaining six nests, all eggs in the clutch showed characteristic honeyguide puncture-holes as frequently observed in unmanipulated nests [4].

**Figure 2.**
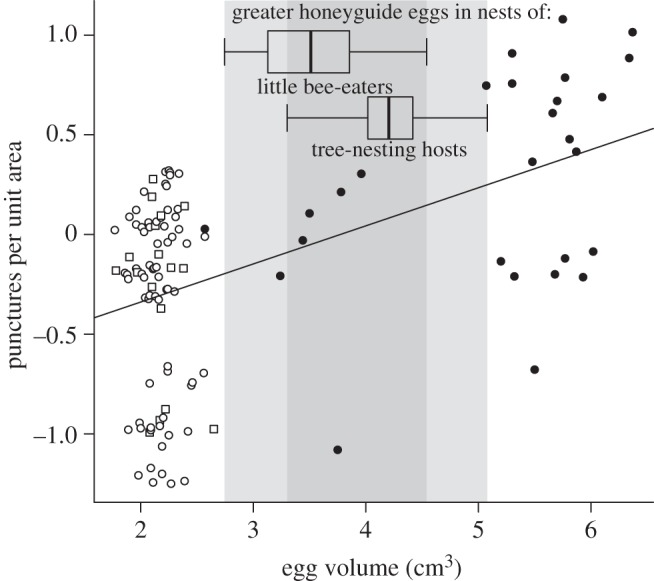
Punctures per unit area (fitted values from mixed model taking into account clutch membership, shell thickness and shape) of control, experimental and host eggs in relation to their volume. The shaded areas show the range of values for real honeyguide eggs (tree-nesting host data from [6]). Squares, control; filled circles, experimental; open circles, host.

On average across all nests, experimental eggs received significantly more punctures than host or control eggs (23.5 ± 2.7 versus 11.4 ± 1.5 and 6.6 ± 2.1, respectively; unequal variances *t*-tests on ranked data: *t* > 4.76, *p* < 0.001). Conclusions were unchanged when analysing punctures per unit surface area and taking shell thickness of the experimental egg and clutch membership into account (egg identity effect: *F*_2,86_ = 4.72, *p* = 0.011; shell thickness effect: *F*_1,25_ = 0.01, *p* = 0.93). The egg identity effect arose because experimental eggs were punctured more frequently than control (*t*_86_ = −2.73, *p* = 0.008) or host eggs (*t*_86_ = −2.59, *p* = 0.011). Differences in puncture rate arose primarily from egg size, because when log-transformed egg volume and shape (breadth/length) were substituted for egg identity, only volume predicted variation in puncture rate (volume effect: *F*_1,86_ = 8.84, *p* = 0.004; shape effect: *F*_1,86_ = 0.40, *p* = 0.53). Figure 2 shows that puncture rate was high for eggs corresponding in volume to greater honeyguide eggs of other sympatric host-races, and greatest for eggs larger than those of any greater honeyguides.

## 4. Discussion

This study has shown that (i) little bee-eater hosts of the greater honeyguide do not react to the presence of a foreign egg that differs from their own in size and shape, and (ii) laying female greater honeyguides do react to the presence of a large foreign egg in bee-eater nests they are parasitizing, by preferentially puncturing it. This suggests that discriminatory egg puncturing by laying female honeyguides imposes a significant selection pressure on previously laid honeyguide eggs. Selection for smaller egg size in greater honeyguides therefore appears more likely to have arisen from interference competition among parasites, than from coevolution with host defences.

Further to eliminating conspecific competitors, puncturing behaviour by greater honeyguides reduces how many host hatchlings their chick needs to kill [4,8] and may allow incubation to continue if their egg is laid late relative to the host clutch [8]. It might perhaps also provide an additional cue of prior parasitism if it reveals the greater shell thickness of another parasitic egg (table 1), although thickened eggshells could also be a defence against multiple parasitism as suggested for other egg-puncturing brood parasites such as cowbirds and *Clamator* cuckoos [7]. Why do honeyguides not heavily puncture all eggs regardless of their dimensions? Presumably, puncturing is tempered by costs, which remain untested; excessive puncturing might directly trigger host desertion [8] or increase a laying honeyguide's chances of detection by hosts (see below). If multiple parasitism exacerbates puncturing behaviour, this would provide an additional route through which parasitic competition can promote virulence [12].

Why do bee-eaters not react to foreign eggs? This is puzzling because interactions between honeyguides and bee-eaters probably have a long evolutionary history, given that the matriline of greater honeyguides parasitizing bee-eaters diverged over 3 Ma from a sister lineage parasitizing various tree-nesting host species [6]. Bee-eaters commonly desert nests visited by a honeyguide ([4] and this study), frequently also ejecting the entire clutch and sometimes re-using the burrow. This suggests that hosts might respond to cues other than presence of a foreign egg, such as heavily punctured eggs or the sight of an adult honeyguide. Clearly, any such defences are frequently breached, so it remains puzzling that bee-eaters have not evolved tactile egg discrimination comparable with that of their parasites or to that of a cowbird host which recognizes eggs on the basis of breadth [13].

In summary, when multiple parasitism is high, interference competition might be an important selective force on avian brood parasites, as it is in, for example, bacteria and helminths [12]. Parasitic competition might thus promote virulence and influence egg size, as honeyguides adapt to avoid the same grisly fate that they impose upon their hosts. If greater honeyguides behave similarly when parasitizing other hosts, competition might also shape host-specific mimicry and select against host switches between ancient lineages of host specialists [6].

## Data accessibility

Data are deposited in the Dryad repository: doi:10.5061/dryad.k6s7h [14].

## References

[1] Davies NB 2000 Cuckoos, cowbirds and other cheats. London, UK: T and A D Poyser

[2] Davies NB Brooke MdeL 1988 Cuckoos versus reed warblers: adaptations and counteradaptations. Anim. Behav. 36, 262–284 (doi:10.1016/S0003-3472(88)80269-0)

[3] Langmore NE Kilner RM 2009 Why do Horsfield's bronze-cuckoo *Chalcites basalis* eggs mimic those of their hosts? Behav. Ecol. Sociobiol. 63, 1127–1131 (doi:10.1007/s00265-009-0759-9)

[4] Spottiswoode CN Koorevaar J 2012 A stab in the dark: chick killing by brood parasitic honeyguides. Biol. Lett. 8, 241–244 (doi:10.1098/rsbl.2011.0739) 21900311 10.1098/rsbl.2011.0739 PMC3297377

[5] Isack HA 1987 The biology of the greater honeyguide *Indicator indicator* with emphasis on the guiding behaviour. DPhil thesis, University of Oxford, Oxford, UK

[6] Spottiswoode CN Stryjewski KF Quader S Colebrook-Robjent JFR Sorenson MD 2011 Ancient host-specificity within a single species of brood parasitic bird. Proc. Natl Acad. Sci. USA 108, 17 738–17 742 (doi:10.1073/pnas.1109630108) 10.1073/pnas.1109630108 PMC3203796 21949391

[7] Brooker MG Brooker LC 1991 Eggshell strength in cuckoos and cowbirds. Ibis 133, 406–413 (doi:10.1111/j.1474-919X.1991.tb04589.x)

[8] Spottiswoode CN Colebrook-Robjent JFR 2007 Eggshell puncturing by the brood parasitic greater honeyguide and potential host counteradaptations. Behav. Ecol. 18, 792–799 (doi:10.1093/beheco/arm025)

[9] Hoyt DF 1979 Practical methods of estimating volume and fresh weight of bird eggs. Auk 96, 73–77

[10] Paganelli CV Olszowka A Ar A 1974 The avian egg: surface area, volume, and density. Condor 76, 319–325 (doi:10.2307/1366345)

[11] Christians JK 2002 Avian egg size: variation within species and inflexibility within individuals. Biol. Rev. 77, 1–26 (doi:10.1017/S1464793101005784) 11911371 10.1017/s1464793101005784

[12] Mideo N 2009 Parasite adaptations to within-host competition. Trends Parasitol. 25, 261–268 (doi:10.1016/j.pt.2009.03.001) 19409846 10.1016/j.pt.2009.03.001

[13] Mason P Rothstein SI 1986 Coevolution and avian brood parasitism: cowbird eggs show evolutionary response to host discrimination. Evolution 40, 1207–1214 (doi:10.2307/2408948) 10.1111/j.1558-5646.1986.tb05745.x 28563499

[14] Spottiswoode CN 2013 Data from: a brood parasite selects for its own egg traits. Dryad Digital Repository. (doi:10.5061/dryad.k6s7h) 10.1098/rsbl.2013.0573 PMC3971702 23966598

